# Automated quantification of proliferation with automated hot-spot selection in phosphohistone H3/MART1 dual-stained stage I/II melanoma

**DOI:** 10.1186/s13000-016-0484-4

**Published:** 2016-04-09

**Authors:** Patricia Switten Nielsen, Rikke Riber-Hansen, Henrik Schmidt, Torben Steiniche

**Affiliations:** Department of Pathology, Aarhus University Hospital, Nørrebrogade 44, DK-8000 Aarhus C, Denmark; Department of Oncology, Aarhus University Hospital, Nørrebrogade 44, DK-8000 Aarhus C, Denmark

**Keywords:** Computer-assisted image analysis, Immunohistochemical double staining, Hot spot, Melanoma, Phosphohistone H3, Prognosis, Proliferation index

## Abstract

**Background:**

Staging of melanoma includes quantification of a proliferation index, i.e., presumed melanocytic mitoses of H&E stains are counted manually in hot spots. Yet, its reproducibility and prognostic impact increases by immunohistochemical dual staining for phosphohistone H3 (PHH3) and MART1, which also may enable fully automated quantification by image analysis. To ensure manageable workloads and repeatable measurements in modern pathology, the study aimed to present an automated quantification of proliferation with automated hot-spot selection in PHH3/MART1-stained melanomas.

**Methods:**

Formalin-fixed, paraffin-embedded tissue from 153 consecutive stage I/II melanoma patients was immunohistochemically dual-stained for PHH3 and MART1. Whole slide images were captured, and the number of PHH3/MART1-positive cells was manually and automatically counted in the global tumor area and in a manually and automatically selected hot spot, i.e., a fixed 1-mm^2^ square. Bland-Altman plots and hypothesis tests compared manual and automated procedures, and the Cox proportional hazards model established their prognostic impact.

**Results:**

The mean difference between manual and automated global counts was 2.9 cells/mm^2^ (*P* = 0.0071) and 0.23 cells per hot spot (*P* = 0.96) for automated counts in manually and automatically selected hot spots. In 77 % of cases, manual and automated hot spots overlapped. Fully manual hot-spot counts yielded the highest prognostic performance with an adjusted hazard ratio of 5.5 (95 % CI, 1.3–24, *P* = 0.024) as opposed to 1.3 (95 % CI, 0.61–2.9, *P* = 0.47) for automated counts with automated hot spots.

**Conclusions:**

The automated index and automated hot-spot selection were highly correlated to their manual counterpart, but altogether their prognostic impact was noticeably reduced. Because correct recognition of only one PHH3/MART1-positive cell seems important, extremely high sensitivity and specificity of the algorithm is required for prognostic purposes. Thus, automated analysis may still aid and improve the pathologists’ detection of mitoses in melanoma and possibly other malignancies.

## Background

The mitotic index of thin primary melanomas (≤1 mm) was included in the Cancer Staging Manual of the American Joint Committee on Cancer in 2010 [1]. Its recommended quantification in hot spots (1 mm^2^ with most mitoses) by microscopy of H&E stains [2] is, however, very laborious and associated with low intra and interobserver variability [3–6]. Henceforth, prognostic impact of the alternate immunohistochemical proliferation markers Ki67 and phosphohistone H3 (PHH3) have been widely studied [7–12]. Because they both label all types of proliferating cells, recent studies demonstrate great utility of immunohistochemical double stains that combine Ki67 or PHH3 with the melanocytic marker MART1 [6, 10, 12–14]. This enables definite distinction of proliferative melanocytic cells and, e.g., proliferative lymphocytes or endothelial cells.

In accordance with a similar study of nodular melanoma [8], we recently demonstrated that proliferation measured by PHH3/MART1 stains was a much stronger prognostic marker than both Ki67/MART1 and H&E stains in stage I/II melanoma. Moreover, quantification in hot spots was highly superior to quantification in the global tumor area for prognostic purposes [10]. Even though PHH3/MART1 eased the detection of tumor-cell proliferation substantially compared with H&E, manual counting and hunts for a single mitosis were still very cumbersome and time-consuming, and hot-spot selection somewhat subjective. To ensure a manageable workload and repeatable measurements in modern pathology, fully automated quantification including automated hot-spot selection by image analysis seems favorable.

To date, automated quantification of PHH3 single stains has only been reported for the global tumor area in a few malignancies, predominantly breast cancer [15, 16], and very recently for manually selected hot spots in melanoma [11]. Surprisingly, we found no studies evaluating the agreement between manual and automated PHH3 counts, merely their association [17]. Algorithms for automated hot-spot selection are, so far, only reported for Ki67 single stains in adrenal cortical cancer [18], ovarian adenocarcinoma [19], and glioma [20]. Seemingly, all remain untested in context to their clinical purpose. Double stains may, nevertheless, increase accuracy of both automated PHH3 quantification and automated hot-spot selection; especially in melanoma that often contains a prominent lymphatic infiltrate with many proliferative lymphocytes. In addition, double stains enable calculation of various new index types, e.g., based on the exact area of melanocytic cells, rather than just the previously reported number per outlined area. Thus, cellular density that may vary considerably per unit area may more easily be incorporated.

This study aims to present the most optimal quantification of tumor-cell proliferation in PHH3/MART1-stained melanomas. Both manual and fully automated assessments are utilized and compared in hot spots and global tumor areas.

## Methods

### Specimens

Formalin-fixed, paraffin-embedded tissue from 153 primary cutaneous melanomas from the Departments of Pathology at Aarhus University Hospital and Randers Hospital, Denmark, were included [10]. The patient characteristics of this previously described prospective patient cohort with consecutive stage I/II patients from 1997 to 2000 are presented in Table 1. The median Breslow thickness was 1.20 mm (range, 0.24–16 mm) and median age 51 years (range, 23–79 years). Recurrent disease accoured in 43 patients (28 %), in which, three were thin melanoma. The median follow-up time was 12 years (range, 8–14 years) for patients with event-free melanoma [10, 21].

**Table 1:** Patient characteristics

Feature	No. of patients (%)
Breslow thickness (mm)	
≤1	56 (37)
1.01-2.00	53 (35)
2.01-4.00	24 (16)
> 4.00	11 (7)
Unclassified	9 (6)
Ulceration	
Yes	37 (24)
No	116 (76)
Histological subtype	
Superficial spreading	108 (71)
Nodular	32 (21)
Other	13 (8)
Sex	
Male	76 (50)
Female	77 (50)

Prior to start, the Central Denmark Region Committee on Biomedical Research Ethics approved the study.

### Immunohistochemistry

BenchMark XT (Ventana Medical Systems, Inc., Tucson, AZ, USA) performed new PHH3/MART1 stains on 3-μm sections because stains of former study were inadequate for image analysis due to both very weak and dark MART1 staining [10]. In short, polyclonal rabbit antibody Phospho-Histone H3 (Ser10; 1:300; 32 min; Cell Signaling Technology, Inc., Danvers, MA, USA) and monoclonal mouse antibody MART-1 (M2-7C710, 1:50; 40 min; Cell Marque Corporation, Rocklin, CA) were applied in sequence and visualized by Ventanas Detection Kits: ultraView Universal 3,3’-Diaminobenzidin (DAB) and ultraView Universal Alkaline Phosphatase Red. Positive controls were included. Slides were counterstained with Mayer’s hematoxylin and bluing reagent.

### Scanning

**Fig. 1:**
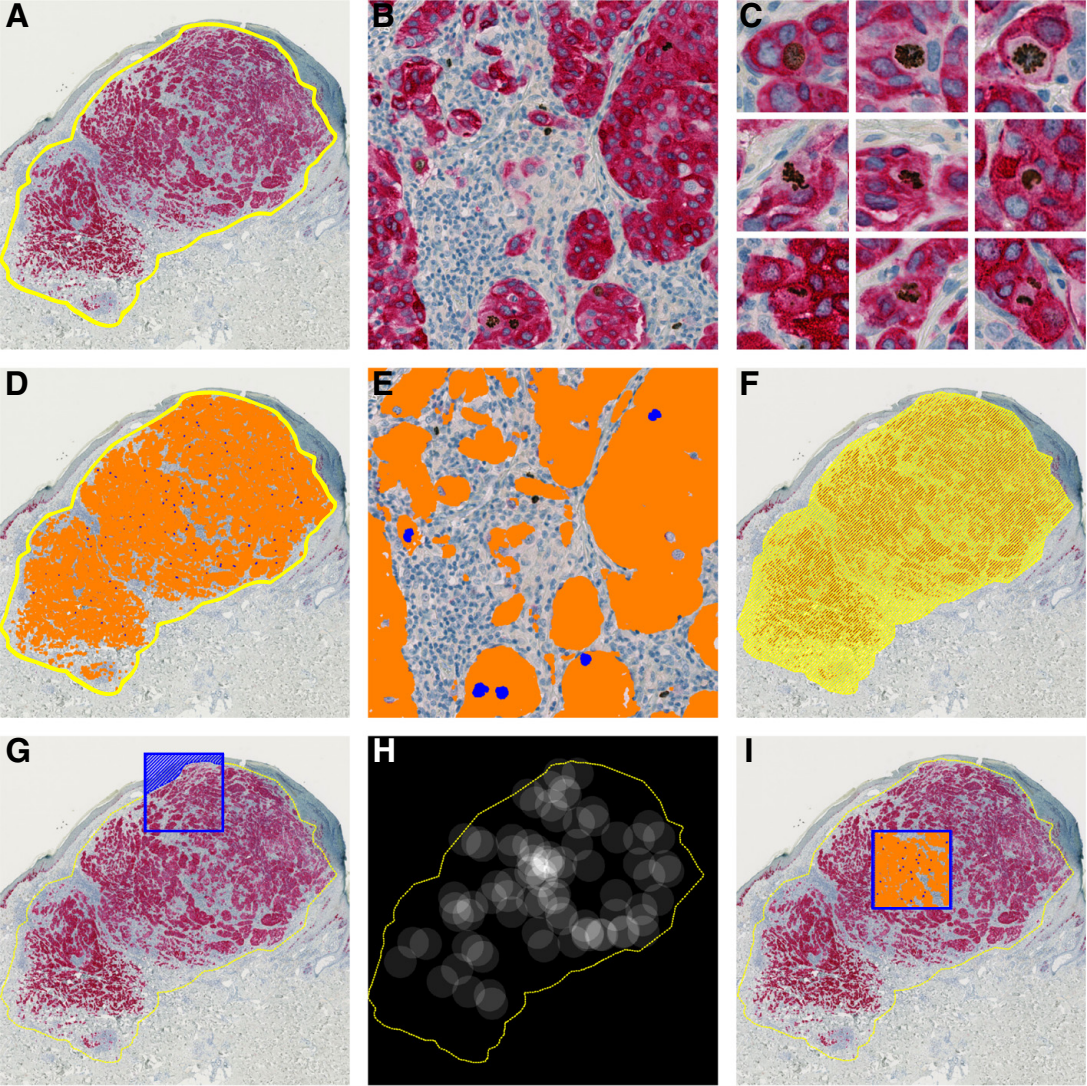
Superficial spreading melanoma stained for PHH3/MART1. **a** Manual outline of global tumor (yellow line). **b-c** PHH3/MART1-positive cells with further display of cells in late G_2_ and M: prophase, metaphase, anaphase, and telophase. **d-e** Automated analysis within outline; MART1-verified tumor area includes orange and blue labels of MART1 and nuclei, respectively. **f** Area within global tumor outline. **g** Manual hot spot excluding epidermis. **h** Automated heat map. **i** Automated hot spot.

Whole slide images were captured by Nanozoomer 2.0HT (Hamamatsu Phototonics K.K., Hamamatsu City, Japan) at magnification 20X (Fig. 1a-c).

### Manual quantification

The global dermal tumor area (Fig. 1a) was outlined manually in Visiopharm Integrator System 5.0.1.1122 (Visiopharm A/S, Hørsholm, Denmark). PHH3/MART1-positive cells, defined as nuclei more brown than blue with noticeable MART1 surrounding (Fig. 1c), were counted manually at optical magnification 40X within the global outline and in a manually selected hot spot, i.e., a fixed 1-mm^2^ square where epidermal regions and skin adnexa were omitted (Fig. 1g), if necessary. Previous comparison of one big 1-mm^2^ hot spot versus four small adjoining 0.25-mm^2^ hot spots within dermis (similar to the conventional method) revealed no distinct difference in their prognostic impact [10]. All manual procedures were performed without knowledge of patient outcome.

### Automated quantification

In the Visiomorph DP module (Visiopharm), the brown, blue, and red staining colors were highlighted by color deconvolution, in which a standard deviation filter further enhanced the contours of PHH3-positive nuclei. In addition, the intensity band of the HSI (hue, saturation, intensity) color model highlighted PHH3 positivity. Thresholding functions partitioned the image into the image classes brown PHH3, red MART1, and blue hematoxylin. In addition to color intensity, post-processing algorithms further highlighted PHH3-positive and PHH3-negative cells based on size, MART1 surrounding, color intensity, and nuclear irregularity. One particular step included dilation of PHH3; hence, cromatin of especially the anaphase and telophase was fused into one object. Blue nuclei of PHH3-negative melanocytic cells were lastly changed into the image class MART1 (Fig. 1d-f), because the reference space was defined as MART1-verified tumor area including nuclei.

Based on the labels of image analysis, a hot spot was automatically detected using the processing step *Object Heat Map*, in which circles identify and cover the object of interest to produce a heat map according to their cluster (Fig. 1h). Accordingly, the center of a circle (radius, 200 μm) was positioned over every PHH3/MART1-positive cell. Within the circle, the value 1 was added, and outside the circle, 0 was added. The hot-spot center was then defined as the pixel position with most circle overlaps, which returns the highest summation or intensity value. This point was encompassed by a 1-mm^2^ square (Fig. 1h and i). Epidermal regions and skin adnexa were manually omitted, if necessary. Only the hottest hot spot was selected; however, more than one hot spot can, in theory, occur. Numerical outcome of such should, nevertheless, be identical or almost identical (minor regions may be subtracted from the 1-mm^2^ reference area) when merely reporting the number per square.

### Index calculations

MART1-adjusted indices were defined as the number of PHH3/MART1-positive cells divided by the MART1-verified tumor area including nuclei within the outlined global tumor area (Fig. 1d and e) and a hot spot. Hot-spot indices were also reported as merely the number within the hot-spot square.

### Statistical analyses

Stata 12.0 (StataCorp, College Station, TX, USA) performed analyses where two-sided *P*-values less than 0.050 were considered statistically significant.

**Fig. 2:**
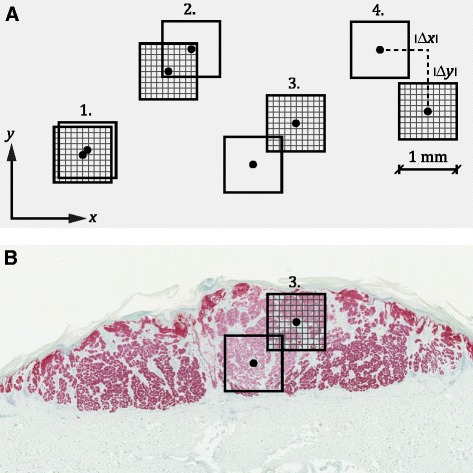
Categorical comparison of manual and automated hot spots. **a** Sketched examples of the 1-mm^2^ hot spots of each method (hatched or unhatched squares) within the four categories: 1. *Perfect overlap*; 2. *Mayor overlap*; 3. *Minor overlap*; 4. *No overlap*. Categorization was based on absolute distances between the center coordinates of the two squares, that is, |Δ*x*| and |Δ*y*|. **b** Display of *minor overlap* of manual and automated hot spots in melanoma with MART1-verified tumor area of 4.80 mm^2^

Bland-Altman plots and Wilcoxon signed-rank tests compared manual and automated procedures. Paired *t* tests compared the location (*x* and *y* coordinates) of hot-spot centroids in image. In addition, the frequency of manual and automated hot spots that overlapped was established based on the distances between both the *x* and *y* coordinates of the square centers (see Fig. 2). The degree of overlap was divided into the categories: *perfect overlap* (distances, < 0.10 mm), *mayor overlap* (distances, 0.10–0.74 mm), *minor overlap* (distances, 0.75–1.0 mm), and *no overlap* (distances, > l.0 mm).

Univariate and multivariate analyses were performed using the Cox proportional hazards model. The group with low PHH3 was the reference group. The multivariate model included the index categorized by its median, the Breslow thickness (log-transformed), and ulceration. Time to recurrent disease was calculated from the time of diagnosis, and patients who either were alive without recurrence at last clinical follow-up or died without evidence of melanoma were censored.

## Results

**Fig. 3:**
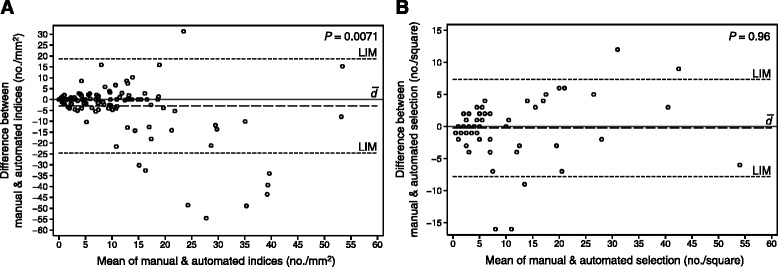
Comparison of the manual and automated index (**a**) and hot-spot selection (**b**). **a** Bland-Altman plot of manual and automated PHH3 counts in the global MART1-verified tumor area; three outliers with differences of -332, -125, and 259 cells/mm^2^ were excluded. **b** Bland-Altman plot of automated PHH3 counts in manually and automatically selected hot spots; two outliers with differences of -21 and 40 cells were excluded. In both plots, indices of manual procedures are subtracted from automated procedures. Mean difference between indices ($\overline{d}$), 95 % limits of agreement (LIM), and *P*-value of Wilcoxon signed rank tests are shown

Global manual and automated PHH3 counts are compared in Fig. 3a, and their ability to detect PHH3/MART1 positivity is outlined in Table 2. The Spearman correlation coefficient was 0.77 (*P* < 0.0010).

**Table 2:** Comparison of manual and automated detection of PHH3/MART1-positive cells

		Automated, no. (%)		Total, no. (%)
		Count = 0	Count ≥ 1	
Manual, no. (%)	Count = 0	41 (27)	14 (9)	55 (36)
	Count ≥ 1	6 (4)	92 (60)	98 (64)
Total, no. (%)		47 (31)	106 (69)	153 (100)

The automated counts of the manually and automatically selected hot spots in cases with coinciding PHH3/MART1 positivity (*n* = 92) are compared in Fig. 3b. The Spearman correlation coefficient was 0.86 (*P* < 0.0010).

The mean difference between *x* coordinates of the manual and automated hot-spot selection was 0.058 mm (95 % CI, -0.30–0.42 mm, *P* = 0.75) and -0.31 mm (95 % CI, -0.62–0.0060 mm, *P* = 0.054) for *y* coordinates. Coordinates of the hot-spot centroids were perfectly identical in 22 cases (24 %), with mayor overlap in 35 cases (38 %), with minor overlap in five cases (5 %), and without overlap in 30 cases (33 %).

The prognostic ability of the different PHH3 quantification types is displayed in Table 3 for univariate analysis and in Table 4 for multivariate analysis. The ability to detect recurrent disease is outlined in Table 5 for fully manual and fully automated hot spots.

**Table 3:** Univariate Cox regression analysis

PHH3 quantification type with index categorized by median	Progression-free survival		
	HR	95 % CI	*P*
Manual count & outline, hot spot, 0 vs. ≥ 1.0	14	3.4 - 58	> 0.0010
Manual count & outline, MART1-adjusted hot spot, ≤ 7.7/mm^2^ vs. > 7.7/mm^2^	6.1	2.7 - 14	> 0.0010
Manual count & outline, MART1-adjusted global, ≤ 3.3/mm^2^ vs. > 3.3/mm^2^	4.3	2.1 - 9.0	> 0.0010
Automated count & manual outline, hot spot, 0 vs. ≥ 1.0	3.4	1.7 - 6.8	> 0.0010
Automated count & outline, hot spot, ≤ 2.0 vs. > 2.0	3.8	2.0 - 7.1	> 0.0010
Automated count & outline, MART1-adjusted hot spot, ≤ 14/mm^2^ vs. > 14/mm^2^	3.3	1.6 - 6.5	0.0010
Automated count & manual outline, MART1-adjusted global, ≤ 4.0/mm^2^ vs. > 4.0/mm^2^	2.5	1.3 - 4.9	0.0060

*Abbreviations*: *PHH3* phosphohistone H3, *HR* hazard ratio, *CI* confidence interval

**Table 4:** Multivariate Cox regression analysis including Breslow thickness (continuous, log-transformed) and ulceration

PHH3 quantification type with index categorized by median	Progression-free survival		
	HR	95 % CI	*P*
Manual count & outline, hot spot, 0 vs. ≥ 1.0	5.5	1.3 - 24	0.024
Manual count & outline, MART1-adjusted hot spot, ≤ 7.7/mm^2^ vs. > 7.7/mm^2^	3.0	1.2 - 7.1	0.015
Manual count & outline, MART1-adjusted global, ≤ 3.3/mm^2^ vs. > 3.3/mm^2^	1.9	0.82 - 4.3	0.14
Automated count & manual outline, hot spot, 0 vs. ≥ 1.0	1.9	0.92 - 3.8	0.085
Automated count & outline, hot spot, ≤ 2.0 vs. > 2.0	1.3	0.61 - 2.9	0.47
Automated count & outline, MART1-adjusted hot spot, ≤ 14/mm^2^ vs. > 14/mm^2^	1.7	0.81 - 3.7	0.16
Automated count & manual outline, MART1-adjusted global, ≤ 4.0/mm^2^ vs. > 4.0/mm^2^	1.3	0.62 - 2.7	0.49

NOTE. Only patients with a classified Breslow thickness were included (*n* = 144). Thus, the number of events was reduced to 39.

*Abbreviations*: *PHH3* phosphohistone H3, *HR* hazard ratio, *CI* confidence interval

**Table 5:** Diagnostic performance of fully manual and fully automated hot-spot quantification

	Manual hot spot (cut-off, 1 cell per square)		Automated hot spot (cut-off, 2 cells per square)	
		95 % CI		95 % CI
Sensitivity, %	95	84 - 99	67	52 - 81
Specificity, %	48	39 - 58	71	62 - 79
ROC area	0.83	0.76 - 0.90	0.74	0.65 - 0.83
Positive predictive value, %	42	32 - 52	48	35 - 61
Negative predictive value, %	96	88 - 100	85	76 - 91

*Abbreviations*: *ROC* receiver-operating characteristic, *CI* confidence interval

## Discussion

The principle finding of the study was that automated and manual results were very comparable for both counting cells and selecting hot spots. The prognostic impact of automated quantification in the clinically preferred hot spot was, however, noticeably reduced compared with manual quantification. Algorithms may, nevertheless, still serve as valuable tools for pathologists, and the hot-spot detection seems easily addable to other malignancies and stains.

**Fig. 4:**
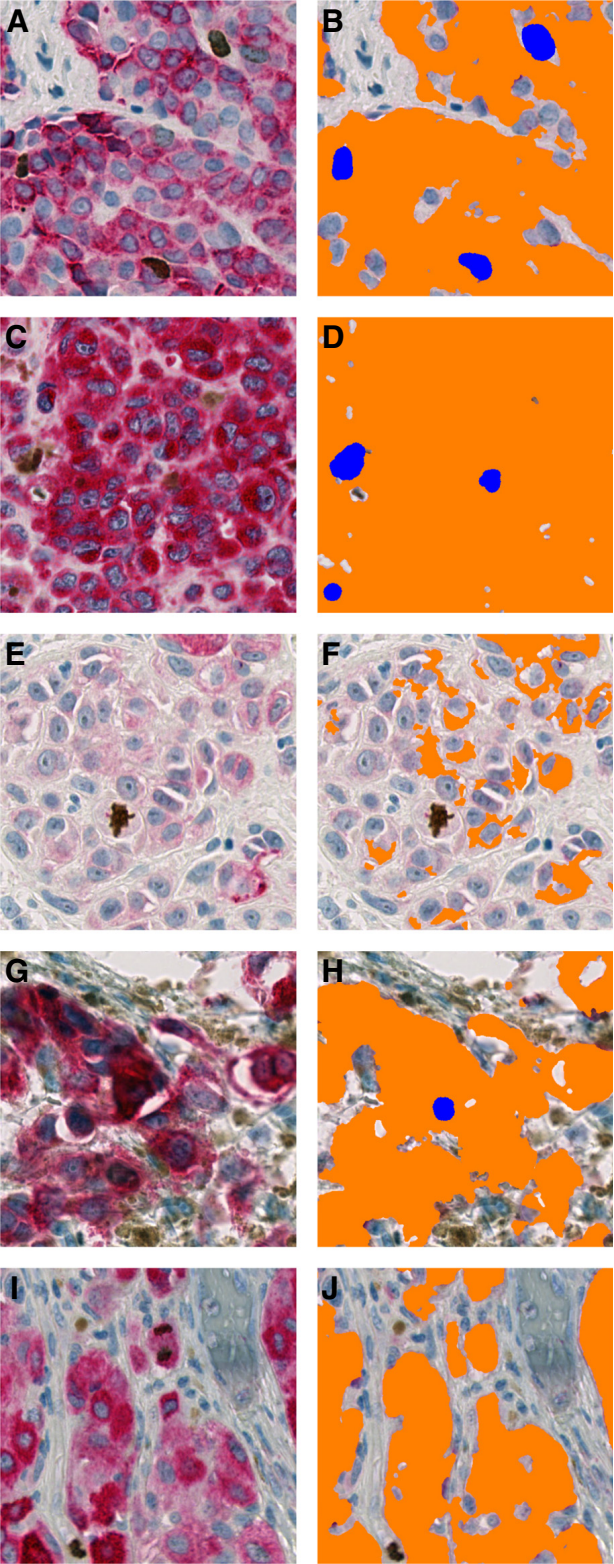
Errors of automated PHH3/MART1 image analysis in five different melanomas. **a** Three PHH3-positive non-melanocytic cells (possibly lymphocytes) that overlay or are in close connection to MART1. **b** They are thus falsely counted as PHH3/MART1-positive cells. **c** Brown pigment embedded in or in close connection to MART1. **d** False-positive counts consequently occur. **e** Very pale MART1 stain of melanocytic cells. **f** A PHH3-positive mitosis is thus overlooked. **g** Very dark MART1 stain or artifact that resembles pixel intensities of brown staining. **h** Because shape, in addition, resembles a nucleus, a false-positive count is made. **i** Telophase of PHH3/MART1-positive cell. **j** Distance between chromatin of dividing nucleus is too large to fuse them into one object with the post-processing step *dilation*, and this mitosis is therefore overlooked

Although automated indices, in general, were systematically higher than manual indices, global counts of PHH3/MART1-positive cells were remarkably comparative in most cases (Fig. 3a, Table 2), and their prognostic impact were, accordingly, in fairly the same range (Tables 3 and 4). Only 33 cell counts (22 %) differed with more than five cells per mm^2^. They were primarily lesions with a very small MART1-verified tumor area (median, 0.39 mm^2^); hence, the misinterpretation of a single cell may alter the calculated index substantially. This was the case for 12 lesions where absolute counts actually only differed with one to four cells. In the remainder of cases, the automated algorithm was unsuccessful because of overlaying PHH3-positive lymphocytes (*n* = 7; Fig. 4a and b), heavy pigmentation (*n* = 6; Fig. 4c and d), pale MART1 (*n* = 5; Fig. 4e and f), or very dark MART1 that overlap pixel intensities of PHH3 (*n* = 3; Fig. 4g and h).

Only a few other groups have presented an automated quantification of PHH3, but without our improved accuracy of immunohistochemical double staining [11, 15, 16, 22, 23]. Even fewer have compared their automated and manual cell counts [11, 15, 16], and apparently the actual agreement of methods remains unreported [17]. In a study of melanoma, the association between manual and automated counts was strong by Pearson’s correlation, and their prognostic impact comparable; however, the difference between mean counts seems large (approx. 6/mm^2^ for manual counts vs. 16/mm^2^ for automated counts) [11]. Our numbers were in the same range (7/mm^2^ vs. 10/mm^2^), but the mean difference considerably smaller. Previous automation of Ki67/MART1 stains has demonstrated usefulness in both diagnosis and prognosis of melanoma [10, 24, 25], but fundamental differences between stains exclude extrapolation of results. The Ki67/MART-positive cells are very abundant, which elevates cut-off point, and cells are mostly circular because they often are in the G_1_, S, or G_2_ phases rather than mitosis. This eases image analysis because algorithms that enhance circular structures may be utilized [25].

Because the prognostic impact of hot spots seems highly superior to global analysis [10] (Tables 3 and 4), an automated selection was developed. The fully automated and fully manual selection was very similar, and hot spots overlapped in most cases (67 %). In cases without overlap (*n* = 30), manual and automated cell counts were often in the same range (only nine differed with more than five cells). After reevaluation of these nine cases, mistakes pertained to heavy pigmentation (*n* = 5), overlaying PHH3-positive lymphocytes (*n* = 2), pale MART1 (*n* = 1), and general failure of the algorithm’s cluster circles to depict the correct hot spot (*n* = 1). After the manual and automated hot-spot selection, an automated count was performed for both hot-spot types. The automated algorithm selects the hot spot with cluster circles based on labels of the preceding automated image analysis. If correctly selected, the subsequent automated count of the automatically selected hot spot should always be higher or equal to the automated count of the manually selected hot spot. This was the case in 65 of 92 lesions (71 %; Fig. 3b). In the remainder cases, the cluster circles failed because another hotter hot spot actually existed. Yet, the difference between counts was very small (median, three cells per hot spot), and most hot spots actually still overlapped (*n* = 19). Nevertheless, few errors must be expected in automated hot-spot selection, which mostly pertains to the chosen radius of the cluster circles. Underlining their comparability, the prognostic performance of the manual and automated selection was very alike (Tables 3 and 4). No distinct parallel was observed between lesion size and hot-spot overlap, but hot spots were of course more likely to completely overlap in very small lesions. The mean MART1-verified tumor area was 2.69 mm^2^ (range, 0.020–50 mm^2^) for the entire cohort.

Because no previous study has developed or evaluated automated hot-spot selection in PHH3 stains or evaluated the currently presented algorithms for Ki67 stains [18–20] in a clinical setting, studies supporting our comparability of manual and automated selection are warranted.

It has previously been established that the global MART1-verified tumor area is preferable to simply using the area within the global outline, which includes surrounding stroma, as reference space in melanoma diagnosis (Fig. 1d vs. Fig. 1f) [24], and this was also the case for prognostic purposes in this study (data not shown). This is possibly explained by lesional dependent amounts of stromal tissue that also may vary according to the subjective outline of the observer. Surprisingly, the MART1-adjusted hot spot of manual counts was quite inferior to just reporting the number of PHH3/MART1-positive cells within the square (Tables 3 and 4); hence, cellular density seems of less importance in melanoma hot-spot analysis compared with global analysis. The result may, however, just reflect the fact that an overall division into *PHH3-positive* and *PHH3-negative* patients is more important for prognostic purposes.

Despite our concordance between manual and automated indices and hot spots, the prognostic impact of automated quantification in a hot spot was noticeably reduced compared with the fully manual quantification (Tables 3 and 4). This is possibly because of the study’s low cut-off point (one cell per square for fully manual hot spots), which divides the cases into either *PHH3-positive* or *PHH3-negative*; hence, misinterpretation of only one cell is of grave importance in the cases with no vs one PHH3/MART1-positive cell (*n* = 68). High sensitivity and specificity of both manual and automated analysis is thus required. Because the manual count was gold standard, all lesions were very carefully examined by virtual microscopy, many repeatedly, and one could speculate whether some mitoses would have been left undetected in a routine setting with a heavy workload. In automated analysis, similar high performance is difficult to achieve because a few errors inevitable will occur (Fig. 4). Primarily, it seems difficult to prevent false-positive counts of PHH3-positive non-melanocytic cells or brown pigment surrounded by MART1 and false-negative counts of tumor cells that express no or only sparse MART1 (Fig. 4a-f). In addition, proper recognition of PHH3/MART1-positive cells seems especially difficult because the shape of PHH3-positive nuclei changes throughout mitosis, and MART1 has a tendency to become pale and sometimes almost white around the nuclei (Fig. 1c) for unknown reasons. Furthermore, the anaphase and telophase may also occasionally be overlooked or regarded as two rather than just one cell (Fig. 4i and j), even though an algorithm may be designed to fuse the chromatin into one object. Inevitably, staining quality also changes despite standardization. The inability of algorithms to deal with variations in staining color and intensity between and within sections is, in general, a mayor drawback of digital image analysis. An algorithm that normalizes signals of immunohistochemical stains, e.g., by control sections, is thus highly needed. Other chromogenes than DAB or the use of virtual double stains could, in addition, be explored to enhance the results of automated image analysis in melanoma.

Still, all our lesions were analyzed with the same fixed protocol; hence, manual review with alteration of algorithms or rejection of result may possibly improve prognostic impact noticeably, and this is possibly a general necessity in the establishment of diagnostic digital pathology.

Though the prognostic impact of our fully automated quantification of PHH3 in hot spots diminished, algorithms may still serve as valuable tools for the pathologist. Adhering to the current staging manual where presence of a single H&E mitosis alters the tumor category and henceforth the subsequent surgical procedures (sentinel lymph node biopsy) in thin melanoma, recognition of one PHH3/MART1-positive cell is important. Although PHH3 highlights mitotic figures substantially compared with H&E, careful and time-consuming screening may still frequently be needed. Given its high sensitivity, the labels or heat map of the global index quantification may vividly direct the observer’s attention to cells that are most likely in mitosis, and the risk of overlooking that single mitotic cell may possibly be reduced. Furthermore, automated analysis with automated hot-spot selection may prove very useful in other malignancies or stains with higher clinical cut-off points than melanoma. For instance, in early breast cancer, a PHH3 cut off of 13 cells per 1.8 mm^2^ has been proposed to distinguish high and low risk patients [15], and a Ki67 index above 30 % was recently acknowledged as an indicator for adjuvant chemotherapy [26]. In addition, neuroendocrine tumors are divided into well- or poorly differentiated by a Ki67 cut off of 20 % or a hematoxylin-eosin mitotic index of 20 mitoses per 2 mm^2^; however, the suggested cut offs for PHH3 are as low as four cells per 2 mm^2^ [27].

## Conclusions

In conclusion, manual detection of PHH3/MART1-positivity in a 1-mm^2^ hot spot yielded an outstanding prognostic performance in our study of stage I/II melanoma; however, adjusting the reference space for tumor cellularity and automation of quantification reduced its prognostic impact noticeably. The automated index quantification and automated hot-spot selection were, nevertheless, very comparable to their manual counterpart. The automated results may thus still aid and improve the pathologist’s detection of mitoses in melanoma and possibly be useful in other malignancies and future research studies. Differentiation into merely *PHH3/MART1-positive* or *PHH3/MART1-negative* cases was ideal for prognostic purposes in our cohort, but further studies are warranted to establish the optimal cut-off point for thin melanoma.
